# Bridging the gap: exploring the impact of bootcamp on non-technical skills and professional development in early-career orthopaedic trainees

**DOI:** 10.1186/s12909-025-07740-4

**Published:** 2025-08-27

**Authors:** Zaha Kamran Siddiqui, Christopher Lewis, Antonia Myatt, Raveen Jayasuriya, Vivek Balachandar, Tim Herrick, James Tomlinson

**Affiliations:** 1 Manchester University NHS Foundation Trust, Manchester, UK; 2 Mid Yorkshire Hospitals NHS Trust, Wakefield, West Yorkshire UK; 3 University of Sheffield, Sheffield, South Yorkshire UK; 4 Sheffield Teaching Hospitals NHS Foundation Trust, Sheffield, South Yorkshire UK; 5 Barnsley Hospital NHS Foundation Trust, Barnsley, South Yorkshire UK

**Keywords:** Professionalism, Education, Psychological adaptation, Social adjustment, Orthopaedic, Professional identity formation

## Abstract

**Background:**

Surgical education faces numerous challenges, including reduced training opportunities and the need for both technical and non-technical skills development. This study explores how a surgical bootcamp for new Trauma and Orthopaedic (T&O) trainees can facilitate the development of Non-Technical Skills for Surgeons (NOTSS) and form a foundation for ongoing professional development during the critical transition to specialty training.

**Methods:**

Using a mixed-methods approach, we examined the impact of a two-day bootcamp for T&O ST3 trainees (*n* = 15) in the Yorkshire region. Data collection included pre/post-bootcamp questionnaires (100% response rate) and semi-structured interviews at two weeks (*n* = 11, 73%) and six months (*n* = 6, 40%) post-bootcamp. Analysis followed Braun and Clarke’s thematic approach to identify patterns across participants’ experiences.

**Results:**

Four major themes were identified: Integration & Expectations, Perception and Application of NOTSS, Psychological Safety & Learning Culture, and Building Relationships. The bootcamp functioned as a transitional space facilitating adaptation across social, psychological, and professional domains. Trainees progressed from initial skepticism about NOTSS to recognizing their importance in clinical practice. However, a notable disconnect persisted between their conscious recognition of these skills’ value and their predominantly unconscious application in daily practice, suggesting that awareness alone did not automatically translate to deliberate practice. The bootcamp provided foundations for professional socialization through peer networks, senior mentorship, and creating psychological safety, which collectively contributed to professional development.

**Conclusions:**

Even brief surgical bootcamps can serve as valuable experiences that support trainees’ integration into specialty training through multiple adaptation processes. The early introduction of NOTSS plants important concepts that trainees apply in practice, even without explicit recognition. By addressing social and psychological aspects of training alongside technical and non-technical competencies, bootcamps can be conceptualized as foundations for ongoing professional development rather than isolated skills workshops. Future educational interventions should consider integrating structured follow-up to help trainees consciously identify and develop non-technical competencies.

**Supplementary Information:**

The online version contains supplementary material available at 10.1186/s12909-025-07740-4.

## Introduction

Surgical education faces unprecedented challenges as training paradigms evolve in response to modern healthcare demands. Work hour restrictions resulting from the European Working Time Directive have intensified scrutiny of both training adequacy and patient outcomes [1]. Additionally, structural shifts in training pathways due to COVID-19 and recovery efforts have collectively contributed to diminishing opportunities for hands-on surgical experience [2]. While the reduction in clinical exposure directly impacts the development of technical skills, it also has broader implications for the acquisition of crucial non-technical competencies. Consequently, many trainees report feeling underprepared for the responsibilities of higher surgical training and consultant practice [3]. 

Recent educational theory suggests that effective surgical training requires attention not only to technical proficiency but also to professional development processes, proposing that beyond demonstrating competencies (‘Does’), practitioners must internalize professional values to truly embody their professional role (‘Is’) [4]. The Royal College of Surgeons of Edinburgh has formalized non-technical skills for surgeons (NOTSS) through a dedicated curriculum co-developed by behavioural psychologists, surgeons, and anaesthetists with expertise in risk management across high-reliability industries. This framework categorizes these competencies into four distinct domains: leadership, teamwork and communication, decision-making, and situational awareness [5]. These non-technical competencies, though less visible, play an essential role in surgical practice and patient outcomes [6]. 

This emphasis on NOTSS acquisition also aligns with evolving perspectives on professional competence, social assimilation and psychological development which serve as necessary foundations for professional identity formation [7]. This process of professional socialization involves both acquiring skills and becoming integrated into the profession [8]. The importance of this integration is highlighted by the General Medical Council, which has noted that early-career difficulties among surgeons frequently stem from deficits in interpersonal and cognitive skills rather than technical incompetence [9]. Further reports from professional organizations consistently highlight teamwork failures and unprofessional behaviours as persistent issues undermining both patient safety and workplace culture [10, 11]. Despite the recognized importance of NOTSS through recent curricular innovations such as ‘Capabilities in Practice’ [12], NOTSS development remains largely consigned to the hidden curriculum—tacitly acquired through observation and experience rather than through explicit instruction—leaving many trainees without structured opportunities to cultivate these essential competencies [13]. 

Surgical bootcamps have emerged as potential solutions to address these early training deficits. The term has been borrowed from military fitness bootcamps and has been defined as intensive, focused training using experiential learning and hands-on practice to learn new skills and knowledge in a safe environment [14]. While traditionally conceived as platforms for technical skill acquisition [15, 16], growing evidence suggests bootcamps serve a broader educational function by creating environments conducive to professional socialization, mentorship, and non-technical skills development for ongoing professional development of trainees [14]. This potential aligns with sociocultural learning theories, particularly with concept of “communities of practice,” where professional identity and competence develop through legitimate participation in professional activities alongside peers and more experienced practitioners [8]. 

Trainees experience liminality at career transitions [17] and most bootcamps play a dual role - teaching trainees’ technical knowledge to accelerate their settling in and ease the challenge of the transition. These perspectives suggest that bootcamps may facilitate not only technical skill development but also the social and professional adaptation necessary for ongoing professional development of trainees. Our bootcamp brought this liminality and discomfort to the forefront. By making these challenges explicit and focusing on them primarily, we wanted to explore if we could help trainees manage this transition, whilst also exposing them to non-technical skills that have been shows to change patient outcomes [18, 19]. 

Building on these perspectives, we developed an analytical framework to interpret our findings, proposing that successful integration into specialty training requires adaptation across three interconnected domains: social, psychological, and professional. Social adaptation involves the process of professional socialization involving both acquiring skills and becoming integrated into the professional community [8]. Psychological adaptation encompasses cognitive and emotional adjustments to new roles and responsibilities [20]. Professional adaptation refers to the acquisition and application of both technical and non-technical competencies required for effective practice [21]. The intersection of these domains create the foundation which may contribute to trainees’ professional development and evolving sense of self as surgical professionals [4, 22]. While the bootcamp was not explicitly designed around these domains, our analysis revealed that its effects naturally mapped to these adaptation processes, providing a useful lens through which to understand trainees’ experiences.

This study examines one such bootcamp intervention (Table 1) designed for early-career Trauma and Orthopaedic (T&O) trainees in the Yorkshire region. Rather than focusing on the specifics of the intervention itself, our investigation centres on understanding how this brief but intensive experience influenced trainees’ development and application of NOTSS over time.

**Table 1:** Case description - Yorkshire T&O bootcamp

The Yorkshire T&O bootcamp was a 2-day intensive training experience for new ST3 trainees developed in response to feedback about disconnected half-day teaching sessions. Key features included:
SETTING: Non-clinical venue (Leeds University) chosen to reduce hierarchical barriers
TIMING: Second week of training program
FORMAT: Primarily trainee-led sessions with consultants present only when essential
CONTENT: (Additional file 1 for full programme)
• Non-technical skills: Human Factors & NOTSS, leadership development, professionalism
• Technical skills: Common fracture management, spinal emergencies, external fixation
• Training insights: Maximizing training opportunities, curriculum requirements
• Social component: Faculty-organized dinner and informal networking

## Methods

### Study design and aim

This study aimed to explore the impact of a surgical bootcamp (described in Box 1) on new T&O trainees’ development of non-technical skills and their integration into specialty training. To address this aim, we employed a mixed-methods approach with emphasis on qualitative exploration to capture trainees’ experiences and perceptions at multiple time points (2 weeks and 6 months post-bootcamp) [23]. This design allowed us to investigate both immediate and longitudinal impacts of even a brief educational intervention, guided by the following research questions:

1. How does the bootcamp facilitate trainees’ transition into the specialty training programme?
2. What are trainee perceptions of non-technical skills following the bootcamp?
3. To what extent are trainees able to apply their learning on non-technical skills in clinical practice?
4. How does the bootcamp experience influence trainees’ professional development?
5. What aspects of the bootcamp experience beyond formal curriculum contribute to trainees’ professional development?

Although the bootcamp included both technical and non-technical skills training, this study focuses exclusively on understanding of non-technical skills training in bootcamps. The rationale for this focus is twofold: first, the delivery of technical skills in bootcamp settings and trainee perceptions of these skills have already been extensively explored in the existing literature. Second, the interviews provided a wealth of data related to non-technical skills, allowing for a deeper exploration of their impact on professional development.

### Participants

All new T&O trainees (ST3 level) joining the Yorkshire training programme in 2019 were invited to participate in the study (*n* = 15). All participants were provided with a comprehensive information sheet via email before the bootcamp, and written informed consent was obtained in person on the first day.

### Data collection

We collected data at three time points:

1. Pre-bootcamp questionnaires assessed baseline attitudes and confidence levels in technical skills and professional behaviours/NOTSS (Additional file 4).
2. Post-bootcamp questionnaires (within 24 h) measured changes in self-reported competency using a 6-point Likert scale (1 = strongly disagree, 6 = strongly agree) (Additional file 4).
3. Semi-structured interviews at two time points:
  ◦ 2–3 weeks post-bootcamp (11 participants, 73% response rate).
  ◦ Six months post-bootcamp (6 participants, 40% response rate).

The questionnaires served as a preliminary assessment of immediate reactions to the bootcamp (Kirkpatrick level 1–2) [24], while the interviews provided richer data on trainees’ experiences and the longer-term impact on their clinical practice. Each participant was assigned a number (P1-15) to ensure anonymity, and no personally identifiable data was collected.

To refine the topic guide (Additional file 3), pilot interviews were conducted with three candidates selected at random. These were reviewed by the research team, and no modifications were deemed necessary, leading to retention of the original format. Interviews were conducted individually to minimize social desirability bias [25] and were not time-limited, concluding once all relevant topics had been discussed.

### Data analysis

All interviews were digitally recorded, anonymised, transcribed verbatim by an experienced medical secretary, and returned to participants for member checking before analysis. Data were imported into NVivo12 for management and coding [26]. An inductive thematic analysis approach as outlined by Braun and Clarke [27]. The research team first read the transcripts multiple times to become familiar with the data. We then systematically coded meaningful segments of text throughout the dataset. These initial codes were grouped together based on similarity and relevance to the research questions. The coded data were discussed with the wider research team, allowing us to refine our interpretations and group codes into narrower categories. Through this iterative process of coding, discussion, and refinement, we developed a set of themes and subthemes that represented participants’ experiences and perceptions. This collaborative approach enabled us to challenge individual interpretations and develop a more robust thematic framework.

### Reflexivity

The research team acknowledged potential biases arising from their dual roles in the research and training environment. Two authors (JT and CL) were involved in designing and implementing the bootcamp, with JT serving as the Educational Programme Director and supervisor to CL, an educational leadership fellow. Additionally, other session facilitators were existing orthopaedic trainees from the region, which could have influenced participant responses due to peer dynamics. To mitigate these biases, all interviews were conducted by an independent interviewer (VB) with no involvement in the bootcamp’s conceptualization. The bootcamp was conceptualized as an experimental model that would be revised or abandoned if ineffective, fostering a position of critical inquiry rather than validation.

Throughout data analysis, the team maintained reflexive awareness of their positionality, including both faculty and trainee perspectives, regularly challenging interpretations and seeking alternative explanations for emerging patterns in the data [28]. This approach aligns with quality criteria for qualitative research, which emphasize transparency about researcher perspectives and their potential influence on the inquiry process [29]. 

## Results

All Orthopaedic trainees (*n* = 15) commencing ST3 training in the Yorkshire & Humber Deanery in 2019 attended the bootcamp, yielding a 100% participation rate in the study. Pre- and post-bootcamp surveys achieved a 100% response rate. Of the 15 participants, 11 (73%) completed the 2-3-week follow-up interview, and 6 (40%) participated in the 6-month follow-up interview. This substantial reduction in participation at the 6-month point represents a limitation in our ability to draw conclusions about longer-term impacts.

**Fig. 1:**
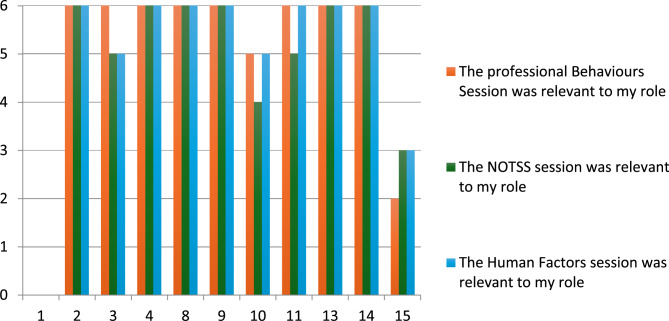
Trainees’ immediate perceptions of non-technical skills during bootcamp

**Fig. 2:**
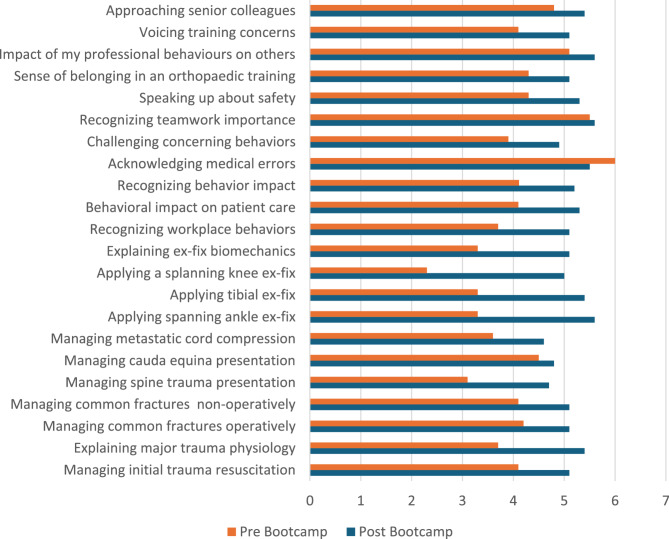
Pre & Post bootcamp scores for behaviours and non-technical skills

Most trainees rated the Professional Behaviours and NOTSS sessions as relevant to their clinical practice (Fig. 1), indicating their initial receptiveness to non-technical skills training. Post-bootcamp questionnaire responses showed improvements in self-reported competencies across both non-technical and technical domains compared to pre-bootcamp baselines (Fig. 2). Trainees reported increased confidence in recognizing key workplace behaviours, addressing patient safety concerns, and seeking senior guidance for training. Notably, pre-existing awareness of teamwork’s importance in surgical care was already strong among participants, as reflected by minimal change in this specific domain.

### Thematic results

Four main themes emerged from our analysis of interviews conducted at 2 weeks and 6 months post-bootcamp: (1) Integration & Expectations, (2) Perception and application of Non-Technical Skills, (3) Psychological Safety & Learning Culture, and (4) Building Relationships.

#### Theme 1: integration & expectations

The bootcamp served as a crucial orientation mechanism for new surgical trainees, establishing expectations and facilitating integration into the training program. At the 2-week point, participants characterized the bootcamp as a “welcoming introduction” that acknowledged their new status and role in the training program. Participant 1 noted, “It’s somebody’s first experience of training, so it’s important to cover the salient bits and feel at home” (2 weeks), while Participant 13 valued the formal recognition of their new position: “It was nice just to have two days acknowledging you are here, you are on the training programme, here is what we expect of you” (2 weeks).

By the 6-month follow-up, trainees had developed a deeper appreciation for how the bootcamp had initiated their integration into the training environment. Participant 4 reflected, “The bootcamp is great for you as a settling point in terms of what’s available to you, what is expected of you as well as meeting the people and building up a relationship and about settling you into the environment. You have to make the effort in deciding how you want to go on with the training and learn” (6 months).

The bootcamp provided a framework for professional expectations and goals, helping clarify training requirements and pathways. Participant 3 highlighted how it offered “clear goals to work towards and a structured way of going about it” (2 weeks), while Participant 1 emphasized its importance for those transitioning between different training structures: “For me, coming from a non-training to training job, it was important to be told this is exactly what you need for Certificate of Completion of Training (CCT) and this is how to achieve this” (6 months).

A significant finding was trainees’ acute awareness of their outsider status upon entering new training environments. Participant 1 observed, “You arrive in a new region, and some people already know each other—that was certainly the case” (2 weeks), while Participant 13 noted their isolation: “At my hospital, senior registrars had been there before, and many juniors had worked in the deanery previously—I was the only brand-new candidate” (2 weeks).

The informal transfer of knowledge emerged as a key mechanism for transitioning from outsider to insider status. Trainees relied heavily on peer networks and senior colleagues to gain insights into training expectations, departmental culture, and unwritten rules. As Participant 13 described, “It was nice to have someone… a couple of years through the system… a bit of advice about things we should do, things we shouldn’t do, things to look out for…. an acknowledgement you are here, this is what to expect, this is what is going to happen” (6 months).

#### Theme 2: perception and application of non-technical skills

The data reveals a complex developmental trajectory for NOTSS. Participants progressed from initial skepticism about NOTSS training: “I was initially a bit surprised these topics were in the bootcamp, but it’s probably the best place,” (P1, 2 weeks) to retrospectively acknowledging the value of this training: “The human factors stuff was good; it puts in your head that you are a registrar and going to have to run things…. In hindsight, it was really quite useful, but you don’t realise it at the time.” (P10, 2 weeks). Participant 8 described the bootcamp as an “ignition” for NOTSS awareness rather than a comprehensive training program.

Participants showed a disconnect between their perceived use of NOTSS and their actual application. Multiple trainees described using these skills “subconsciously” without deliberate implementation. Participant 3 stated, “It’s not something in terms of a conscious thing but I’m sure I’ve used them subconsciously,” while Participant 5 noted “I do not always consciously think about the human factors but if I am doing a ward round, I do try and get all the team members involved.”

The data shows progression from passive observation to active implementation. Early after training, participants primarily described watching consultants react in challenging situations, Participant 8 mentions, “I am just focused on what the consultant is doing and how they are reacting.” Many demonstrated incorporating NOTSS into their own practice, particularly in stress management and team communication in theatres, clinics and during on calls - “I am quite conscious to make sure that I am more measured with my responses if I am feeling stressed” (P14, 6months) and “In clinic, if you respect the nurse you work with then you will get along and have a better rapport rather than wanting to get out and it does affect your performance and patient care” (P5, 6 months).

Self-awareness emerged as a particularly valuable component, with participants highlighting the importance of recognizing their own behaviour in stressful situations: “I find myself thinking about when I come to a stressful situation, like when I am operating and there is a section where I don’t know what to do next and get a little bit more stressed, I figure that I become more quiet and I not tend to ask for help” (P4, 6 months).

The transition from SHO to registrar appeared to involve a significant shift in leadership responsibilities, with delegation identified as particularly challenging by a few participants. Participant 14 noted, “As an SHO you don’t really ever delegate you just get delegated to,” and Participant 1 affirmed, “Giving the SHO some freedom and independence is important, but then also being able to tell them when they’ve not done something is too.” Participants expanded their conception of the surgical role beyond technical competence: “I think I’ve been more receptive to the other roles of a registrar. It’s not just about the operative and getting great numbers. There are lots of other aspects of being a registrar that you can use to influence your or others training” (P1, 6 months).

Despite their experiences, participants suggested that the NOTSS component of the bootcamp was too brief: “I wonder if there is feasibility to run an all-day NOTSS course? The sessions gave a brief overview, but it would be good to cover it in more detail” (P9, 2 weeks). Participant 4 observed that non-technical skills “are always there but nobody really describes them, and I think it is good for reflection and things like that” (P4, 6 months).

#### Theme 3: psychological safety & learning culture

The bootcamp created a supportive learning environment that trainees valued. Participants emphasized how being among peers at similar training stages normalized knowledge gaps. Participant 2 expressed: “You are of similar grade so when they ask you a question you are not looking quite so stupid in front of everyone else” (2 weeks), while Participant 1 acknowledged that “The bootcamp personally for me gave me a bit more confidence” (2 weeks).

This peer-based reassurance was important for trainees who felt isolated in their new environments. Participant 10 highlighted: “The timing of it was good so that you arrive in the following teaching, and you don’t feel like it’s run by ST8s. Especially for someone like me who has come and knows nobody in this deanery. There are other people there that have the same rate of knowledge” (2 weeks).

By the 6-month follow-up, participants reflected on how this early experience had contributed to longer-term confidence. Participant 3 articulated: “It’s where you start in the job, and you feel like am I supposed to be here, am I good enough to be doing this, how I managed to get here—situations where you are a little bit out of depth… I definitely think bootcamp did help me out in terms of as we discussed getting to know everyone and you know what you have got to aim for, and you’ve got a rough timeline of how to get there.”

The data revealed that informal settings significantly enhanced the learning culture. Due to a venue change, one session was relocated to a local pub, creating an unexpectedly productive learning environment. Participant 9 found that “The session (funnily enough in the pub) … was very handy. He talked about portfolio requirements and case based discussions” (2 weeks). Further Participant 10 recounted, “He gave me a tip, for example, fill it out when you are in clinic with your boss, just log in and say, ‘I have filled this out for you do you mind signing it between patients’” (2 weeks).

Similarly, social events fostered relationship-building beyond the clinical or educational context: “The social event on the first evening, it’s always a good excuse to get together… we all started talking to each other outside the work environment” (Participant 7, 2 weeks).

#### Theme 4: building relationships

Professional relationships were a central component of trainees’ experiences, with the bootcamp serving as a catalyst for network formation. At the 2-week timepoint, participants highlighted the immediate establishment of peer support systems: “The guys whipped out their phones… we made an ST3 WhatsApp group. That group has been buzzing” (Participant 1, 2 weeks). These connections became resources for navigating training challenges, with Participant 13 noting, “If you need some info your first port of call is your colleagues, your other ST3… has anyone done this yet?”.

Beyond horizontal peer relationships, vertical relationships with senior trainees provided crucial insights into career progression and professional development. Participant 4 valued that “Senior trainees give us advice on what to expect and what the road looks like a year or two down the line” (2 weeks), while Participant 13 appreciated seniors’ recognition of their transitional status: “My experience of senior colleagues at bootcamp and at work has been excellent—they acknowledge that you are a new registrar and make room for that” (2 weeks).

By the 6-month follow-up, these initial connections with senior colleagues had evolved into deeper mentoring relationships with significant impact on professional identity formation. Participant 4 reflected on the value of consistent relationships: “I really like working with one particular boss for a whole six months… You get to build a relationship with them, know what they like clinically, what changes their decision-making… but also you get a bit of a show aspect in terms of what life could be as a consultant” (6 months). Participant 14 noted, “Looking at how more senior registrars delegate or how they interact with SHOs… you see different leadership styles and start to reflect on what works well and what does not” (6 months).

Trainees’ perceptions of TPDs shifted from hierarchical authority figures to accessible mentors. Participant 4 expressed: “These guys want to know us; they want to know all about how we are getting on… I think they genuinely do have our best interests at heart” (2 weeks), while Participant 1 noted, “I got the impression they genuinely cared about training and wanted to make sure it is a positive experience” (2 weeks). This transformation lowered communication barriers, with Participant 1 acknowledging, “Previously, I might have been worried about contacting them too often… Now, I feel more confident” (2 weeks).

These relationships influenced trainees’ own leadership development. Participant 1 described: “There was a session on how we see managers and how managers see us, which was really interesting… I definitely have a better understanding of the personality traits and pressures they are under. Personally, I have started to be more accessible and reliable with SHOs” (6 months). Participant 3 observed: “I think you take a lot more on board than you realize. Sometimes, I find myself reacting in a situation in a way that I’ve seen senior colleagues handle it” (6 months).

## Discussion

This study aimed to explore the impact of a surgical bootcamp on new T&O trainees’ development of non-technical skills and their integration into specialty training. Our findings suggest that even a brief, two-day bootcamp serves as more than just a technical skills program; it appears to function as a transitional space that facilitates professional socialization, relationship building, and the introduction of non-technical competencies [17]. As one participant described it, the bootcamp provided “a holistic approach and was not just about Orthopaedics.”

The quantitative improvements captured post-bootcamp (Fig. 2) demonstrate enhanced self-reported competencies across all domains. While these represent Kirkpatrick level 1–2 outcomes that have limitations in evidencing sustained behavioural change, they provide initial indication of the bootcamp’s immediate impact on trainees’ confidence and awareness [24]. 

**Fig. 3:**
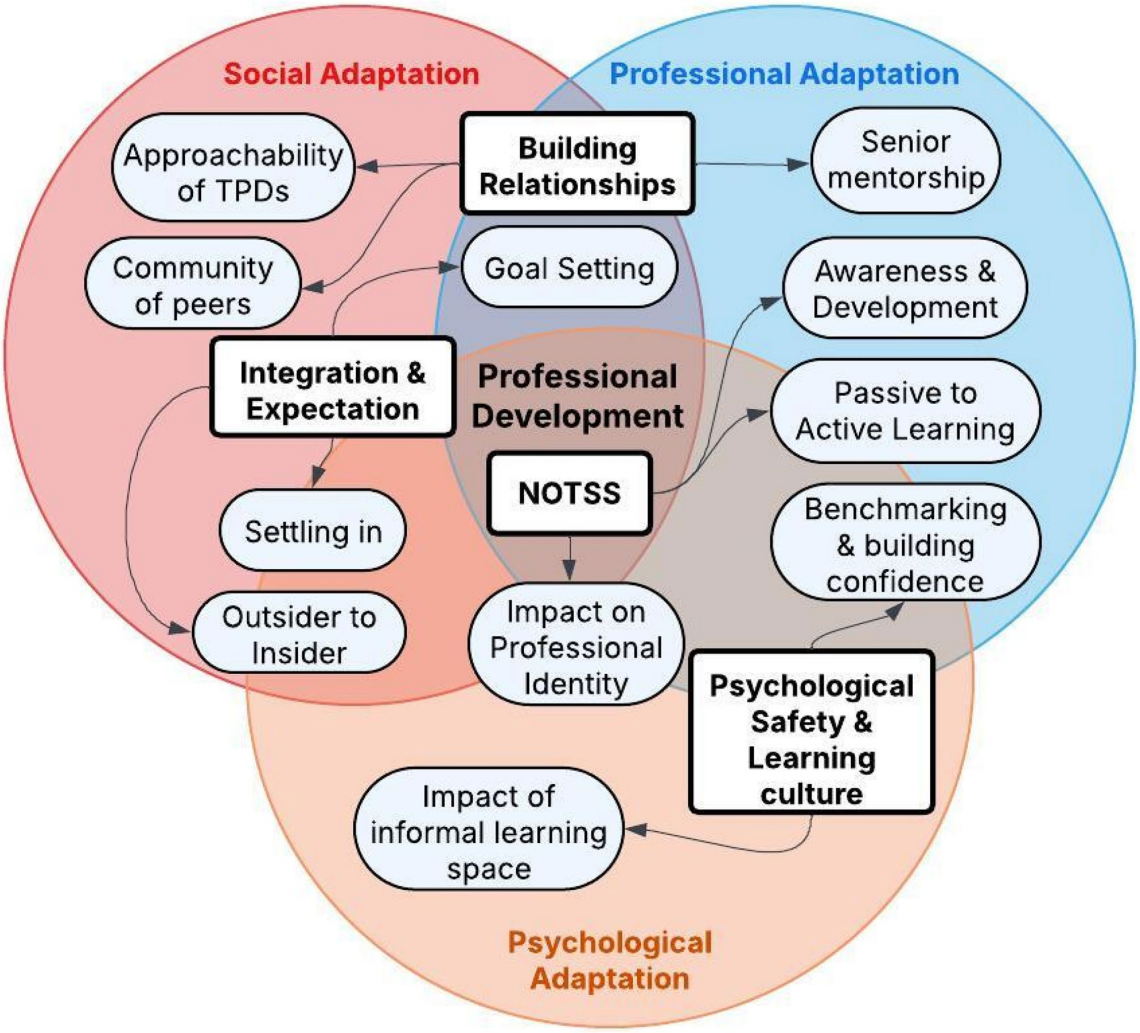
Surgical trainee adaptation framework across social, professional and psychological domains

Our thematic analysis revealed four main themes: Integration & Expectations, Perception and Application of NOTSS, Psychological Safety & Learning Culture, and Building Relationships. These themes can be interpreted through our theoretical framework of adaptation across three interconnected domains: social, psychological, and professional (Fig. 3). This framework aligns with evolving perspectives on professional competence [4, 7] and extends concepts of professional socialization [8] by explicitly recognizing the psychological dimensions of training transitions [21]. The integration of these three domains offers an interpretative lens through which to understand how trainees navigate their transition into specialty training following bootcamp participation, with professional development at its core.

The Integration & Expectations theme illuminates how trainees begin to internalize their new professional roles. As our results showed, participants characterized the bootcamp as a “welcoming introduction” and a “settling point” that facilitated orientation to programmatic expectations. Participant statements such as “It was nice just to have two days acknowledging you are here, you are on the training programme, here is what we expect of you” and “For me, coming from a non-training to training job, it was important to be told this is exactly what you need for CCT and this is how to achieve this” reveal how the bootcamp provided a framework for understanding professional pathways. This transitional process demonstrates strong parallels with the theoretical construct of “legitimate peripheral participation,” wherein novices progressively move from peripheral engagement toward full integration within a professional community of practice [8]. The bootcamp appeared to accelerate trainees’ transition from “outsider” to “insider” status, representing a critical trajectory in professional socialization [17]. 

The perception and application of NOTSS theme reveals the progression in trainees’ understanding and application of non-technical competencies. Our findings revealed a clear evolution from initial skepticism (“I was initially a bit surprised these topics were in the bootcamp”) to retrospective appreciation (“In hindsight, it was really quite useful, but you don’t realise it at the time”). This supports previous research indicating that early-career surgical trainees may not immediately recognize the value of NOTSS training [13]. The trainees’ progression from skepticism to recognition mirrors the professional identity formation process where practitioners move beyond demonstrating competencies (‘Does’) to internalizing professional values (‘Is’) [4]. This transformation aligns with evolving perspectives on professional competence that emphasize the internalization of professional values as a foundation for identity development [7]. Contrary to faculty concerns about overloading trainees with NOTSS content, participants actively requested more training in this area, confirming the assertion that trainees are typically underexposed to these skills [13]. 

This Venn diagram illustrates the interrelationships between major themes from surgical bootcamp (square boxes) and their associated recurring codes (oval boxes) across three adaptation domains: Social, Professional, and Psychological. Each theme contributes to multiple adaptation domains, with key processes (codes) bridging different areas.

Our longitudinal data revealed a significant disconnect between trainees’ conscious perception of their NOTSS use and their actual application. As our results demonstrated, multiple trainees described using these skills “subconsciously” without deliberate implementation yet provided clear examples of application such as “I am quite conscious to make sure that I am more measured with my responses if I am feeling stressed” and “In clinic, if you respect the nurse you work with then you will get al.ong and have a better rapport.” These behavioural modifications represent Kirkpatrick’s level 3 impact (behaviour change) with trainees reporting specific modifications to their clinical practice [24]. This suggests that NOTSS may be integrated into practice through both explicit instruction and implicit modelling; a form of tacit knowledge acquisition that aligns with conceptualization of informal learning in professional contexts [8, 30]. Furthermore, this unconscious implementation reflects how NOTSS development often remains within the hidden curriculum [13], despite curricular innovations like ‘Capabilities in Practice’ [12] that attempt to formalize these competencies.

The bootcamp’s impact extended well beyond its formal curriculum, with informal learning spaces (theme 3) and relationship development (theme 4) emerging as powerful catalysts for professional growth. The Psychological Safety & Learning Culture theme revealed how the peer-structured learning environment facilitated trainees’ confidence. As our results showed, participants consistently emphasized the value of being among peers at similar training stages: “You are of similar grade so when they ask you a question you are not looking quite so stupid in front of everyone else.” This comparative self-assessment without evaluative judgment mitigated feelings of inadequacy frequently documented during transitional periods in medical education. By the 6-month follow-up, this psychological safety had clearly contributed to longer-term confidence, with one participant articulating how the bootcamp helped address impostor syndrome: “It’s where you start in the job, and you feel like am I supposed to be here, am I good enough to be doing this.” This finding aligns with Edmondson’s conceptualization of psychological safety as an essential precondition for effective team learning and professional development [20, 31]. 

Further, the improvement in trainees’ confidence to approach senior colleagues for training advice (Fig. 2) supports our qualitative findings on the transformation of relationships with TPDs from hierarchical to accessible. The development of multifaceted professional relationships—with peers, senior trainees, and TPDs—functioned as conduits for both explicit knowledge transfer and implicit professional socialization. The value trainees placed on these relationships demonstrates Wenger’s ‘communities of practice’ concept where professional identity develops through legitimate participation alongside more experienced practitioners [8]. 

Particularly noteworthy was trainees’ reports of a shift in how they perceived TPDs, with statements such as “These guys want to know us; they want to know all about how we are getting on. I think they genuinely do have our best interests at heart” showing a transformation from viewing them as hierarchical authority figures to accessible mentors. This transformation in how trainees viewed TPDs and subsequently modified their own interactions with juniors exemplifies the professional socialization process underpinning professional identity formation [7] and addresses teamwork failures highlighted by professional bodies [10, 11]. 

Our findings documented how these relationships influenced trainees’ own leadership development. As one participant described six months after the bootcamp: “Personally, I have started to be more accessible and reliable with SHOs,” while another observed unconscious modelling: “Sometimes, I find myself reacting in a situation in a way that I’ve seen senior colleagues handle it.” These relationships made the “hidden curriculum” more accessible, particularly in relation to NOTSS development [32]. Social events and informal settings enhanced knowledge sharing and relationship building, reinforcing the social dimensions of bootcamps [14]. This suggests that NOTSS elements should be intentionally incorporated into educational design rather than treated as supplementary.

### Educational implications

The bootcamp played what participants described as a “transformative role” in accelerating their development of social networks, benchmarking, building confidence, and establishing psychological safety—factors that significantly influenced their perception of the training program itself and inherently contributed to their professional development.

Our findings have several implications for surgical education. First, they suggest that bootcamps should be conceptualized not merely as technical skills interventions but as transitional experiences that can introduce non-technical aspects of surgical training. By doing this and explicitly exploring the challenges of career transitions we can address some the feelings of discomfort and uncertainty at these times.

Second, they highlight the value of incorporating structured NOTSS training early in specialty training, even if its full benefits may not be immediately apparent to trainees. The evolving nature of NOTSS application indicates that single-point interventions are unlikely to be sufficient for comprehensive skills development. Instead, bootcamps should be viewed as foundations for ongoing learning that is reinforced through clinical practice, mentorship, and reflection.

The integration of NOTSS training early in specialty training supports the Royal College of Surgeons of Edinburgh’s formalization of these competencies [5]while acknowledging the challenges in making these skills explicit rather than part of the hidden curriculum [13]. The disconnect between trainees’ unconscious application of NOTSS and their lack of explicit recognition highlights a significant opportunity for further educational interventions. The bootcamp successfully planted NOTSS concepts, yet trainees lacked the structured reflective framework to consciously identify these skills in their daily practice, despite demonstrating capacity for such reflection during research interviews when specifically prompted to consider their non-technical skill development. This gap between unconscious application and conscious recognition suggests an opportunity for structured reflective practice with clinical supervisors, which could bridge the transition from implicit to explicit awareness of NOTSS application in everyday surgical practice. Further, implementing a spiral curriculum approach—where NOTSS concepts are revisited with increasing complexity throughout training—could reinforce these skills and move trainees from unconscious to conscious application. Incorporating NOTSS more formally into workplace-based assessments, similar to technical skills evaluations [12], would provide structured opportunities for reflection and feedback, potentially accelerating development through deliberate rather than incidental practice. Multiple participants reported feeling that the program was “deeply invested in their success,” creating a powerful early impression that shaped their engagement with training. Training Program Directors should consider how elements contribute to the social, psychological, and professional adaptation of trainees, recognizing their intersection as the locus of ongoing professional development.

### Strengths and limitations

This study has several strengths. Its longitudinal design allowed us to capture both immediate perceptions and evolving reflections on the bootcamp experience over a six-month period. The high engagement rate—100% participation in pre- and post-bootcamp surveys and 73% in initial interviews—reflects strong buy-in from the trainee cohort and enhances the credibility of the findings. Additionally, the use of qualitative data collected at two distinct time points offers valuable insight into the sustained influence of even brief educational interventions on trainees’ professional development.

However, there are limitations to consider. The reduced participation at the six-month follow-up (40%) introduces a potential response bias, as later reflections may disproportionately represent more engaged or motivated trainees. While the total number of interviews conducted (*n* = 17) provides a rich dataset, the smaller number of interviews at 6 month follow up limits the breadth of insight into long-term impacts.

Our study focused on a single specialty within one training region, and as such, the findings may not be universally generalisable. Rather than offering a direct implementation template, the aim of this study is to illuminate underlying mechanisms—particularly around identity formation, non-technical skills development, and psychosocial transition—that may be applicable across various contexts.

Recognizing that readers may also be interested in the practical aspects of implementation, we have included additional file 2, which outlines the key principles, logistical considerations, and design decisions that informed our bootcamp delivery. This allows us to share practical insights without detracting from the manuscript’s core focus on educational impact and professional development.

Finally, while our data relies on self-reported perceptions and experiences rather than objective performance measures, this aligns with the qualitative and exploratory nature of our research questions, which aimed to capture how trainees made sense of their development and professional integration following the bootcamp.

### Future directions

Future research should explore the longer-term impact of bootcamps on NOTSS development and professional integration, ideally following trainees throughout their specialty training to completion of training. Comparative studies examining different bootcamp models would help identify the most effective elements for NOTSS development. Additionally, research incorporating objective measures of NOTSS alongside self-reported data would strengthen the evidence base for bootcamp effectiveness. Studies examining how to optimize the timing and integration of follow-up interventions could help address the gap between unconscious and conscious application of non-technical skills that we observed.

## Conclusion

This study suggests that surgical bootcamps may serve as valuable transitional experiences for early-career trainees, supporting their integration into specialty training through multiple adaptation processes. The early introduction of NOTSS appears to plant important concepts that trainees may apply in practice, even without explicit recognition. The development of professional relationships and creation of psychologically safe learning environments seem particularly valuable in facilitating this transition.

These findings indicate that even brief educational interventions can contribute to trainees’ professional development when they address social and psychological aspects of training alongside technical and non-technical competencies. By understanding bootcamps as foundations for ongoing learning rather than isolated skills workshops, educators may more effectively support trainees’ multifaceted adaptation to specialty training.

## Supplementary Information

Supplementary Material 1.

Supplementary Material 2.

Supplementary Material 3.

Supplementary Material 4.

## Data Availability

The bootcamp programme, topic guide for semi-structured interviews, and pre/post bootcamp questionnaires are available in the supplementary files. Additional data generated during this study but not included in the final publication is available from the corresponding author upon reasonable request.

## Abbreviations

NOTSS: Non-Technical Skills for Surgeons
CCT: Certificate if Completion of Training
ST3: Specialty Training
TPDs: Training Programme Directors
T&O: Trauma and Orthopaedic
