# Supplementary material for: Bridging the gap: exploring the impact of bootcamp on non-technical skills and professional development in early-career orthopaedic trainees
Source: BMC Med Educ. 2025 Aug 27;25:1208. doi: 10.1186/s12909-025-07740-4 (PMC12382066; doi:10.1186/s12909-025-07740-4)
Supplement: Supplementary file 1 — Supplementary Material 1. [file 12909_2025_7740_MOESM1_ESM.docx]

**Additional file 1 – Bootcamp Programme**

**Yorkshire Orthopaedic ST3 Boot camp – University of Leeds**

Thursday 10^th^ October

09:30 Introductions / Ground Rules / Question Box

10:00 Workshop 1 - Trauma Physiology (TPD led)

11:00 Coffee

11:15 Workshop 2 – Professional Behaviours (Trainee led)

13:15 Lunch

14:00 Workshop 3 – Non-Technical Skills (Trainee led)

14:45 TPD Welcome (TPD led)

15:15 Coffee

15:30 Workshop 4 – Virtual Fracture Clinic 1 (Consultant led)

16:30 Workshop 5 – Registrar Tips & Tricks + Q&As (Trainee led)

17:00 Finish, Drinks & Dinner

Friday 11^th^ October

09:00 Welcome Back

09:15 Workshop 6 – Virtual Fracture Clinic 2 (Trainee led)

10:15 Workshop 7 – Human Factors (Trainee led)

11:00 Workshop 8 – Spinal Trauma / CES / MSCC (Consultant led)

12:00 Lunch provided by Deanery

13:00 Workshop 9 - Saw bone Ex-Fix (Stryker) (TPD led)

15:30 Q&As + Closing Remarks

16:00 Finish
