# Supplementary material for: Bridging the gap: exploring the impact of bootcamp on non-technical skills and professional development in early-career orthopaedic trainees
Source: BMC Med Educ. 2025 Aug 27;25:1208. doi: 10.1186/s12909-025-07740-4 (PMC12382066; doi:10.1186/s12909-025-07740-4)
Supplement: Supplementary file 2 — Supplementary Material 2. [file 12909_2025_7740_MOESM2_ESM.docx]

**Appendix A: Key Principles for Implementing a Surgical Bootcamp**

To accompany: "Bridging the Gap: Exploring the Impact of Bootcamp on Non-Technical Skills and Professional Development in Early-Career Orthopaedic Trainees"

This appendix outlines our underlying design philosophy, practical considerations, and lessons learned. These principles may be of interest to educators seeking to replicate or adapt bootcamps that support trainees beyond technical competencies, especially during key transitions in training.

**1. Design Philosophy: Bootcamps as Transitional Support**

- Recognising that transitions are inherently challenging
  Stepping into a new role—especially the shift from SHO to registrar—is psychologically and professionally demanding. Many bootcamps attempt to manage this uncertainty by overloading trainees with intense technical and cognitive content.
- Our key principle: Acknowledge the discomfort, don’t hide it
  We asked: *What if, instead of masking the discomfort of transition, we brought it into the open?* By explicitly discussing the challenges and psychological discomfort of “stepping up,” we aimed to normalize these experiences and equip trainees with strategies to navigate them.
- Non-technical skills as central, not supplementary
  We framed the bootcamp around NOTSS (Non-Technical Skills for Surgeons) to help trainees understand their evolving professional roles—not just what they need to do, but who they are becoming.

**2. Practical Implementation Considerations**

- Timing and structure
  The bootcamp took place in the second week of training to allow early peer connections and grounding in the new role. A 2-day format enabled immersion without major disruption to clinical service.
- Venue and setting
  Hosted at a non-clinical venue (Leeds University) to minimize hierarchical cues and promote informal dialogue.
- Content balance
  While our primary focus was on social, psychological, and professional adaptation, we also included some technical content (e.g., fracture management, spinal emergencies). This provided benchmarking opportunities, lent credibility, and met trainees' expectations. However, technical content was not the dominant emphasis.
- Core knowledge for year one success
  We ensured that trainees received clear information on key contacts, programme expectations, and critical milestones (e.g., ARCP timelines, training assessments), which trainees reported as essential for orienting themselves.
- Pre-event planning and coordination
  Implementation required ~4–6 months of lead time, with coordination across training programme directors, rota coordinators, and educational leads. Administrative support was essential for logistics, communication, and delivery.

**3. Lessons and Recommendations**

- Be honest about the transition
  Trainees appreciated the open acknowledgement of the pressures and uncertainties they were facing. Future bootcamps should consider deliberately including content focused on psychological adaptation and identity formation.
- Peer-to-peer delivery model
  The peer-to-peer model fostered a sense of belonging, normalized knowledge gaps, and created space for candid discussions about challenges.
- Ensure senior visibility—but not dominance
  The presence of respected consultants, limited to key sessions and social events, helped reinforce the value of the bootcamp without undermining peer-led delivery.
- Include essential orientation content
  Clear communication around training expectations, timelines, and performance standards helped alleviate anxiety and empowered trainees to take control of their progression.
- Build in credibility and benchmarking
  Including core technical content allowed trainees to benchmark themselves against peers and added legitimacy to the experience, even if not the primary focus.
- Incorporating social components
  Informal interactions, including a faculty-hosted dinner and even a relocated session in a local pub, were intentionally used to foster community and encourage open conversation. These were consistently identified by trainees as high-value aspects of the programme.

**4. Additional Resources**

We have discussed our approach and philosophy further on The Theatre: Surgical Learning and Innovation Podcast: *Registrar Bootcamp*. Available on Spotify: [Registrar Bootcamp – The Theatre](https://open.spotify.com/show/your-link-here) (https://open.spotify.com/episode/0aQtlqB11OfcgwvastbNKM)
