# Supplementary material for: Bridging the gap: exploring the impact of bootcamp on non-technical skills and professional development in early-career orthopaedic trainees
Source: BMC Med Educ. 2025 Aug 27;25:1208. doi: 10.1186/s12909-025-07740-4 (PMC12382066; doi:10.1186/s12909-025-07740-4)
Supplement: Supplementary file 3 — Supplementary Material 3. [file 12909_2025_7740_MOESM3_ESM.docx]

**Additional file 2**

**Interview Questions (Interview Cycle 1):**

Introduction: (Read this to everyone)

An independent interviewer is conducting these interviews, and you are able to speak

freely and honestly. This interview recording will be transcribed and anonymised before

it is made available to the researchers or faculty.

This is the first time the boot camp has been held. The interview questions are deliberately open ended. The style of these questions and the assessment may feel very different to previous educational evaluations. There are no right or wrong answers and I

want you to speak freely and share your thoughts on your experience of the bootcamp. You can speak openly as these interviews are highly confidential

Q1. Tell me about your experience of boot camp

Prompts (only use if needed)

How did it feel to be part of boot camp?

Are there are aspects of the boot camp that have stayed with you since taking part in it?

Did you experience anything other than being taught at boot camp?

What have you found most challenging to incorporate into your work

from boot camp?

What have you found straightforward to incorporate into your work from

boot camp?

What relationships developed at boot camp?

How would you describe the experience of boot camp to a colleague?

Q2. Are there any ways in which this boot camp has influenced how you think or

behave?

Prompts (only use if needed)

How has this changed from your previous thoughts/behaviours?

What happened at boot camp that influenced your thoughts/behaviours?

Did you expect the boot camp might challenge or change your thinking?

Have your boot camp reflections been influenced by your time at work so far?

Q3. Is there anything else you would like to tell us about boot camp?

Prompts (only use if needed)

How did the boot camp compare with what you were expecting?

We hope to meet again in 3 months to ask for further reflections on the

boot camp – is there anything from boot camp that you definitely want to be thinking or

doing three months from now?

**Interview Questions (Interview Cycle 2)**

Introduction: (Read this to everyone)

An independent interviewer is conducting these interviews, and you are able to speak

freely and honestly. This interview recording will be transcribed and anonymised before

it is made available to the researchers or faculty.

This is the first time the boot camp has been held. The interview questions are deliberately open ended. The style of these questions and the assessment may feel very different to previous educational evaluations. There are no right or wrong answers and I

want you to speak freely and share your thoughts on your experience of the bootcamp. You can speak openly as these interviews are highly confidential

Q1. Tell me about your experience of orthopaedic training now you are a few months

in? (Is it what you were expecting after boot camp?)

Prompts (only use if needed)

Is it what you expected?

Are there any things that have been better than expected?

Are there any things that have been worse than expected?

Q2. Looking back to the start of training, what impact did the boot camp have on you

starting out?

Prompts (only use if needed)

What did you find useful about attending the boot camp?

Was there anything you found less useful from the boot camp?

Have any of your thoughts/opinions on the boot camp changed since you

were last interviewed?

If you were designing a boot camp for next year’s new starters, would you

change anything?

Q3. Have you managed to use things you learned at boot camp in your practice?

Prompts (only use if needed)

Have the non-technical skills/Human Factors sessions influenced how you be-

have at work?

Q4. What did you find most helpful about attending a boot camp?

Q5. Is there anything else you would like to tell us about the boot camp and

programme?
