# Supplementary material for: Bridging the gap: exploring the impact of bootcamp on non-technical skills and professional development in early-career orthopaedic trainees
Source: BMC Med Educ. 2025 Aug 27;25:1208. doi: 10.1186/s12909-025-07740-4 (PMC12382066; doi:10.1186/s12909-025-07740-4)
Supplement: Supplementary file 4 — Supplementary Material 4. [file 12909_2025_7740_MOESM4_ESM.docx]

**Additional file 3:**

**T&O ST3 Bootcamp Pre/Post Questionnaire**

**Instructions:** Please rate your agreement with each statement on a scale of 1-6 where 1 = Strongly Disagree and 6 = Strongly Agree.

**Participant Research Number:** _______________

**Professional Skills & Attitudes**

1. The Professional Behaviours session was relevant to my role
2. The Non-Technical Skills for Surgeons (NOTSS) session was relevant to my role
3. The Human Factors session was relevant to my role
4. I feel able to recognise key behaviours in the workplace which enable or inhibit team working
5. I am conscious of how my behaviours may impact directly on patient care
6. I feel able to recognise how my behaviours may impact directly on patient care
7. All doctors can make errors, regardless of their seniority
8. I can be assertive in challenging others concerning behaviours in the workplace
9. Effective teamwork is vitally important in the care of surgical patients
10. I feel able to speak up about patient safety concerns in the workplace
11. I have a sense of belonging in an orthopaedic training programme
12. My professional behaviours have an important impact on others
13. I can speak up about issues or concerns related to my training programme
14. I could confidently approach senior colleagues for advice about training

**Clinical & Technical Skills**

1. I am comfortable managing the initial resuscitation of major trauma patients
2. I can explain the physiological response to major trauma
3. I am comfortable managing common fractures seen in fracture clinic treated operatively
4. I am comfortable managing common fractures seen in fracture clinic treated non-operatively
5. I am comfortable managing the initial presentation and investigation of spine trauma
6. I am comfortable managing the initial presentation and investigation of cauda equina
7. I am comfortable managing the initial presentation and investigation of metastatic cord compression
8. I am comfortable applying a spanning ankle external fixator
9. I am comfortable applying a tibial external fixator
10. I am comfortable applying a spanning knee external fixator
11. I can explain the biomechanical principles of external fixation

**Additional Feedback**

1. Is there anything that you wish had been included in the Bootcamp?
